# Association between the Triglyceride–Glucose Index and Non-Alcoholic Fatty Liver Disease in patients with Atrial Fibrillation

**DOI:** 10.1186/s40001-023-01188-2

**Published:** 2023-09-19

**Authors:** Xiaozhong Li, Fenfang Zhan, Tian Peng, Zhen Xia, Juxiang Li

**Affiliations:** 1 Department of Cardiovascular Medicine, The Second Affiliated Hospital of Nanchang University, Nanchang, 330006 China; 2 Department of Anesthesiology, The Second Affiliated Hospital of Nanchang University, Nanchang, 330006 China

**Keywords:** Triglyceride–glucose index, Insulin resistance, Non-alcoholic fatty liver disease, Atrial fibrillation

## Abstract

**Background:**

The triglyceride and glucose index (TyG), as a surrogate of insulin resistance (IR), is closely associated with non-alcoholic fatty liver disease (NAFLD). However, the association between the TyG index and NAFLD in atrial fibrillation (AF) is unknown. Therefore, the purpose of this study is to explore the association between the TyG index and NAFLD in AF.

**Methods:**

This retrospective study was performed at Nanchang University’s Second Affiliated Hospital. The AF patients who were hospitalized from January 2021 to December 2022 were enrolled. The association between the TyG index and NAFLD in AF patients was assessed by logistic regression and restricted cubic spline analysis. The ability of TyG index for identifying NAFLD was estimated by the area under the receiver operating characteristic (ROC).

**Results:**

In this study, 632 people participated in the final analysis, with 176 (27.84%) having NAFLD. In the full adjustment model, there is an association between the TyG index and NAFLD [per 1 unit increment; odds ratios (ORs): 3.28; 95% confidence interval (CI) 2.14, 5.03]. Compared to the lowest tertile (TyG index < 8.29), the ORs for the highest tertile (TyG index ≥ 8.82) were 4.15 (95%CI: 2.28, 7.53). Dose–response analysis showed that the TyG index and NAFLD have a nearly linear relationship (*P* non-linear = 0.71). The area under the curve (AUC) of the TyG index is 0.735.

**Conclusions:**

Our findings showed a significant association between the TyG index and NAFLD. The TyG index may be a good marker for predicting NAFLD in AF patients.

**Supplementary Information:**

The online version contains supplementary material available at 10.1186/s40001-023-01188-2.

## Introduction

Non-alcoholic fatty liver disease (NAFLD) is characterized by the presence of steatosis in over 5% of hepatocytes and its association with metabolic risk factors (particularly obesity and type 2 diabetes), and it is not caused by drinking too much alcohol (men ≥ 30 g/day and  women ≥ 20 g/day )or other long-term liver diseases [1]. NAFLD represents a spectrum of conditions, including simple steatosis, non-alcoholic steatohepatitis (NASH), fibrosis, and ultimately cirrhosis [2]. NAFLD is found in about 25% of people around the world [3]. It is a major cause of chronic liver disease [3]. In China, the rapid lifestyle transitions contributed to an increase in the prevalence of NAFLD, which was 29.2% [4]. Cardiovascular diseases are the main cause of death for NAFLD patients [5].

NAFLD has adversely affected the cardiac electrical system [6]. There are significant associations between NAFLD and an increased atrial fibrillation (AF) risk [7, 8].AF is the most common arrhythmia, which affects more than 46.3 million people in the world [9]. From a pathophysiology point of view, the association between NAFLD and AF is complex and caused by the interplay of different, bidirectional pathways, including inflammation, and impaired glucose and lipid metabolism [10]. Thus, identifying high-risk groups of NAFLD in patients with AF is of great significance for improving the prognosis of AF. 

Abnormal glucose and lipid metabolism are common in AF patients. Insulin resistance (IR) can promote this biological process [11]. There is a strong IR associated with NAFLD [12]. The TyG index is an IR marker that is consistent with the current gold standard for IR diagnosis (hyperinsulinemic glucose clamp test). [13] Prior research has indicated that higher TyG increases NAFLD risk in the general population [14]. However, the report about AF patients is limited. Therefore, in this study, the purpose is to determine the association between the TyG index and NAFLD risk among AF patients.

## Methods

### Study design and population

This retrospective study enrolled 1561 consecutive AF patients who were hospitalized at Nanchang University's Second Affiliated Hospital from January 2021 to December 2022. Inclusion criteria included: (1) AF patients; (2) participants over the age of 18. Exclusion criteria included: (1) participants below the age of 18; (2) excessive alcohol consumption (≥ 30 g per day for men and ≥ 20 g per day for women); (3) participants with missing fasting triglyceride, glucose, and NAFLD data; and (4) participants with Hepatitis B Virus (HBV) and/or Hepatitis C Virus (HCV) infection [14, 15]. The Second Affiliated Hospital of Nanchang University granted ethical approval for our experiment, which strictly complied with the Declaration of Helsinki (2013) (No. 13, 2023, Nanchang, P.R. China).

### Data collection

We reviewed the electronic medical record to gather the general patient demographic and clinical information. The demographic information includes sex, age, body mass index (BMI), systolic blood pressure (SBP) and diastolic blood pressure (DBP), smoking history, AF type, duration of AF, and chronic disease, which includes hypertension, diabetes mellitus, and dyslipidemia. The clinical data included aspartate aminotransferase (AST), alanine aminotransferase (ALT), total cholesterol (TC), triglyceride (TG), high-density lipoprotein cholesterol (HDL-C), low-density lipoprotein cholesterol (LDL-C), estimated glomerular filtration rate (eGFR), glycated hemoglobin (HbA1C), and uric acid (UA).

### Definitions for the TyG index and HSI

The TyG index and the hepatic steatosis index (HSI) were calculated by applying the following formulae:

A) $${\text{TyG index }} = {\text{ Ln }}\left[ {{\text{TG }}\left( {\text{mg/dL}} \right) \, \times {\text{ fasting glucose }}\left( {\text{mg/dL}} \right)/2} \right]$$ [16].
B) $${\text{HSI }} = \, 8 \, \times {\text{ ALT/AST ratio }} + {\text{ BMI }}\left( { + 2,{\text{ if diabetic}}; \, + 2,{\text{ if female}}} \right)$$ [17].

### Definitions for AF and NAFLD

AF is defined as previously having an AF history or being diagnosed based on electrocardiograph findings, which include irregular f waves with a frequency of 350–600 b.p.m. and an irregular ventricular response [18].

Fatty liver disease was identified by abdominal ultrasound using a 3.5-MHz transducer. NAFLD was determined to be the occurrence of fatty liver without the presence of heavy alcohol consumption (men ≥ 30 g/day, and women ≥ 20 g/day), drugs, or viral-induced steatosis [19].

### Statistical analysis

The continuous variables are expressed as the mean ± standard deviation (SD) for the normally distributed data or the median with an interquartile range for the nonnormally distributed data, whereas the categorical data are shown as frequency percentages. The differences in baseline characteristics by NAFLD status were evaluated using an independent two-sample t-test for continuous variables and a Chi-square test for categorical variables.

The odds ratios (ORs) and 95% confidence intervals (CIs) for NAFLD with TyG index were determined using binary logistic regression analysis. Potential confounding variables include age, gender, AF type, dyslipidemia, diabetes, BMI, eGFR, AST, ALT, HDL-C, UA, duration of AF, hypertension, and smoking. The dose–response of  the TyG index associated with NAFLD was evaluated via restricted cubic spline curves.

Interactions were tested using subgroup analysis and adjusted ORs and 95% CIs were exhibited in forest plot. The subgroup analyses are based on the following predefined variables: sex, age (< 65 vs ≥ 65 years), BMI (< 30 vs ≥ 30 kg/m^2^), current smoking (yes vs no), eGFR (< 90 vs ≥ 90 ml/min/1.73m^2^), hypertension (yes vs no), diabetes mellitus (yes vs no), and dyslipidemia (yes vs no). The subgroup analysis was compared with tertile 3 of the TyG index and tertile 1 to enhance the statistical power. The receiver operating characteristic (ROC) curve was applied to calculate the predictive value of the various indicators  for NAFLD. In all analyses, two-sided *p*-value of < 0.05  were considered statistically significant. All data analyses were performed using R software version 4.1.3 (www.R-project.org) and SPSS software (version 20; IBM Corp., Armonk, NY, USA).

## Results

### Characteristics and parameters of the participants

**Fig. 1:**
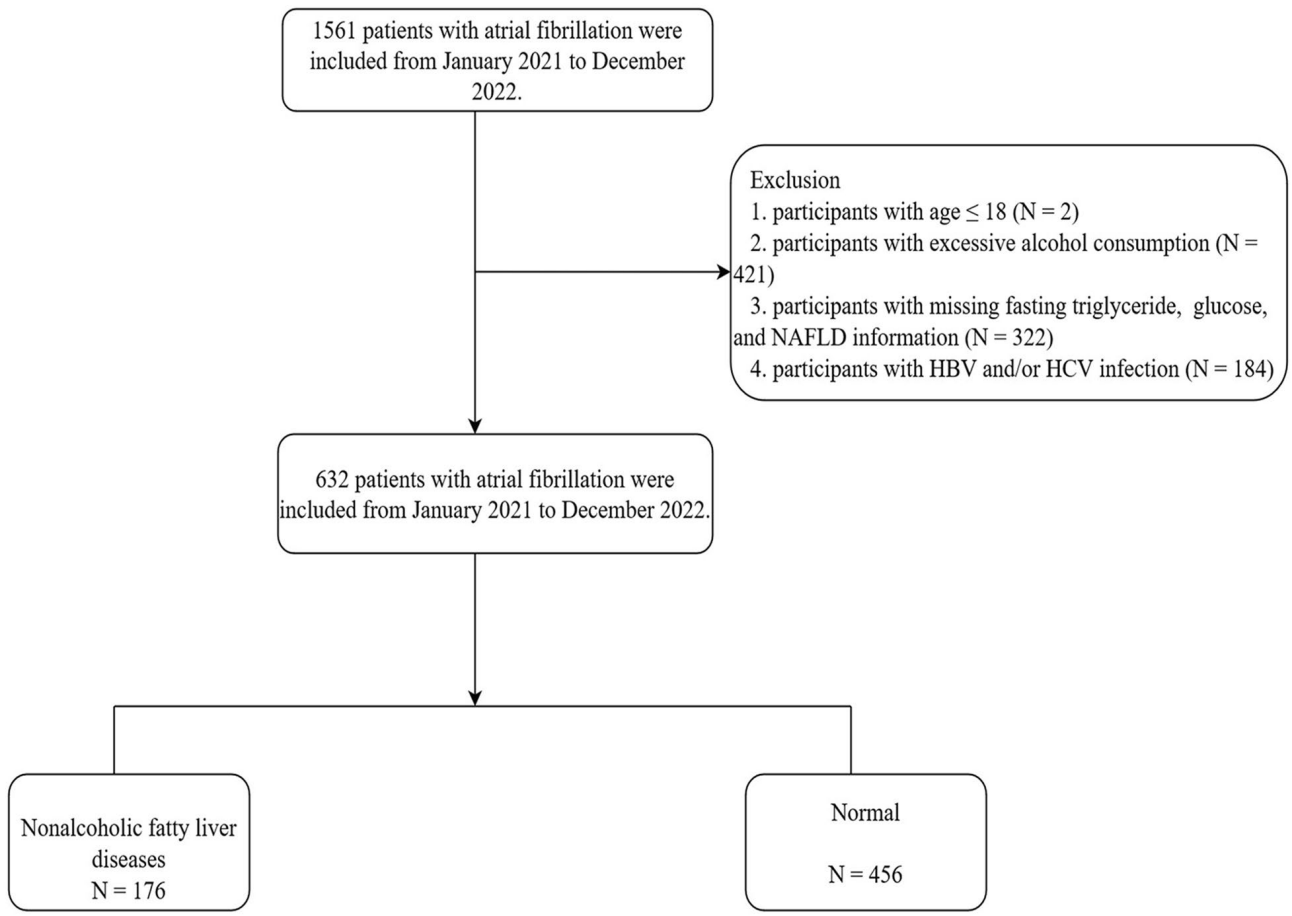
Study flow diagram. *NFLD* non-alcoholic fatty liver disease, *HBV* hepatitis B virus, *HCV* hepatitis C virus

The flow diagram of the study is shown in Fig. 1. From January 2021 to December 2022, we enrolled 1561 patients. Participants under the age of 18 (*N* = 2), current drinkers (*N* = 421), those with missing fasting triglyceride, glucose, and NFLD information (*N* = 322), and those with HBV and/or HCV infection (*N* = 184) were excluded. Eventually, only 632 patients are included in the analysis.

Table 1 provides the clinical characteristics of the study population based on NAFLD status**.** The proportion of NAFLD was 27.84% (176/632). The mean (SD) age in the group with and without NAFLD was 62.8 (10.51) and 65.71 (9.39) years, respectively. Compared to patients in the normal group, those in the NALFD group had higher BMI, SBP, AST, ALT, TC, TG, LDL-C, glucose, HbA1C, UA, and HIS levels. In contrast, the HDL-C level was lower. Meanwhile, diabetes and dyslipidemia were higher (*P* < 0.01). Particularly, NAFLD patients have higher TyG index levels compared to those without the disease (*P* < 0.01).

**Table 1:** Characteristics of the study population

Characteristics	Total (*N* = 632)	NAFLD (*N* = 176)	Normal (*N* = 456)	*P*
Age, year	64.81 (9.81)	62.48 (10.51)	65.71 (9.39)	< 0.01
Female, *n* (%)	269 (42.56)	69 (39.20)	200 (43.86)	0.29
BMI, kg/m^2^	24.26 (3.38)	25.64 (3.69)	23.73 (3.09)	< 0.01
Smoke, *n* (%)	134 (21.20)	34 (19.32)	100 (21.32)	0.47
Persistent AF, *n* (%)	275 (43.51)	76 (43.18)	199 (43.64)	0.92
SBP, mmHg	128 (19)	130 (19)	126 (19)	0.02
DBP, mmHg	73 (14)	77 (14)	76 (14)	0.81
Duration of AF, months	34.31 (48.57)	37.87 (56.42)	32.94 (45.18)	0.72
Laboratory results				
AST, mmol/L	23.52 (19.29–28.71)	24.93 (20.10–30.38)	23.19 (18.93–28.06)	0.04
ALT, mmol/L	20.04 (13.80–28.76)	23.30 (17.01–33.76)	18.73 (13.06–26.53)	< 0.01
TC, mmol/L	4.24 (1.06)	4.59 (1.18)	4.10 (0.98)	< 0.01
TG, mmol/L	1.23 (0.90–1.71)	1.62 (1.11–2.38)	1.14 (0.86–1.53)	< 0.01
HDL-C, mmol/L	1.16 (0.31)	1.08 (0.30)	1.19 (0.32)	< 0.01
LDL-C, mmol/L	2.49 (0.83)	2.78 (0.95)	2.38 (0.76)	< 0.01
Glucose, mmol/L	5.13 (4.62–5.88)	5.62 (4.88–6.89)	5.02 (4.55–5.61)	< 0.01
HbA1C	5.80 (5.50–6.10)	5.90 (5.50–6.45)	5.70 (5.40–6.00)	< 0.01
eGFR, mL/min/1.73m^2^	83.02 (21.65)	85.75 (21.90)	81.95 (21.48)	0.05
UA, mmol/L	377.33 (103.78)	394.59 (104.08)	370.62 (103.00)	< 0.01
HSI	31.51 (4.89)	33.80 (5.61)	30.62 (4.26)	< 0.01
TyG index	8.61 (0.62)	9.00 (0.69)	8.46 (0.53)	< 0.01
Chronic disease, *n* (%)				
Hypertension	359 (56.80)	107 (60.80)	252 (55.26)	0.21
Diabetes	133 (21.04)	63 (35.80)	70 (15.35)	< 0.01
Dyslipidemia	267 (42.25)	108 (61.36)	159 (34.78)	< 0.01

The continuous variables are expressed as the mean (SD) for the normally distributed data or the median with an interquartile range for the nonnormally distributed data. The categorical variables are expressed as numbers (percentages)

*BMI* body mass index, *SBP* systolic blood pressure, *DBP* diastolic blood pressure, *AST* aspartate aminotransferase, *ALT* alanine aminotransferase, *TC* total cholesterol, *TG* triglyceride, *HDL-C* high-density lipoprotein cholesterol, *LDL-C* low-density lipoprotein cholesterol, *eGFR* estimated glomerular filtration rate, *HbA1C* glycated hemoglobin, *UA* uric acid, *TyG* triglyceride–glucose, *HSI* hepatic steatosis index

The basic characteristics of patients by tertiles of the TyG index are presented in Additional file 1: Table S1. Compared to patients in tertile 1 of the TyG index, those in tertile 3 have a younger age, a higher BMI, TC, TG, glucose, LDL-C, HbA1C, lower HDL-C, more NAFLD, diabetes, and dyslipidemia (*P* < 0.05).

### Association of the TyG index with risk of NAFLD

**Fig. 2:**
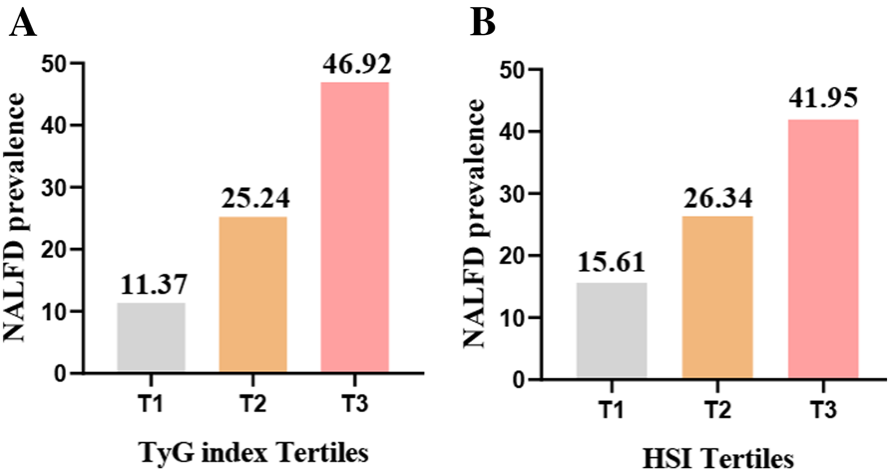
Prevalence of NAFLD based on the tertiles of TyG index (**A**), HIS (**B**). Classification of TyG tertiles: T1 (≤ 8.29), T2 (8.29–8.82), T3 (≥ 8.82); HSI tertile: T1 (≤ 29.26), T2 (29.26–33.13), T3 (≥ 33.13). *NAFLD* non-alcoholic fatty liver disease, *TyG* triglyceride–glucose, *HSI* hepatic steatosis index

The NAFLD prevalence among the tertile 3 of TyG index was 46.92%, which increased 4.13-fold compared to that of the tertile 1 (Fig. 2A). The cut-off for defining IR was set at a TyG index of ≥ 8.76 [20]. The NAFLD prevalence among the IR group was 41.38%, which increased 2.26-fold compared to that of the non-IR group (Additional file 1: Fig. S1). As shown in Table 2, we assessed  the TyG index associated with the risk of NAFLD risk in the crude and adjusted models. The TyG index was significantly associated with NAFLD (per 1 unit increase: OR = 3.27; 95%CI 2.13, 5.02). In the crude model, compared to patients in the tertile 1 of the TyG index, those in tertiles 2 and 3 were significantly associated with NAFLD risk; the ORs were 2.63 (95%CI 1.55, 4.45), and 6.89 (95%CI 4.16, 11.40), respectively. Compared to the patients in the lowest tertiles of the TyG index, the OR (95% CI) for NAFLD was 4.07 (2.24,7.39) in the highest, after additional adjustment for age, gender, AF type, dyslipidemia, diabetes, BMI, eGFR, AST, ALT, HDL-C, UA, duration of AF, hypertension, and smoking.

**Table 2:** Association of the triglycerides–glucose index with risk of non-alcoholic fatty liver diseases

TyG index	Case/N	Crude model OR (95%CI)	*P*	Model I OR (95%CI)	*P*	Model II OR (95%CI)	*P*
Per 1 unit increase	176/632	4.51 (3.21, 6.34)	< 0.001	3.59 (2.40, 5.36)	< 0.001	3.27 (2.13, 5.02)	< 0.001
Tertiles							
T1 (≤ 8.29)	37/211	Ref.	1.0	Ref.	1.0	Ref.	1.0
T2 (8.29–8.82)	46/210	2.63 (1.55, 4.45)	< 0.001	2.54 (1.47, 4.39)	< 0.001	2.22 (1.24, 3.98)	0.007
T3 (≥ 8.82)	93/211	6.89 (4.16, 11.40)	< 0.001	4.97 (2.73, 8.40)	< 0.001	4.07 (2.24, 7.39)	< 0.001
*P* for trend		< 0.001		< 0.001		< 0.001	

Crude model was unadjusted for any factors; Model I was adjusted for age, gender, AF type, dyslipidemia, and diabetes. Model II was adjusted for Model I, BMI, eGFR, AST, ALT, HDL-C, UA, duration of AF, hypertension, and smoking

*95% CI* 95% confidence interval, *OR* odds ratio, *TyG* triglyceride–glucose, *BMI* body mass index, *eGFR* estimated glomerular filtration rate, *AST* aspartate aminotransferase, *ALT* alanine aminotransferase, *HDL-C* high-density lipoprotein cholesterol, *UA* uric acid

### Dose–response relationship between the TyG index and NAFLD

**Fig. 3:**
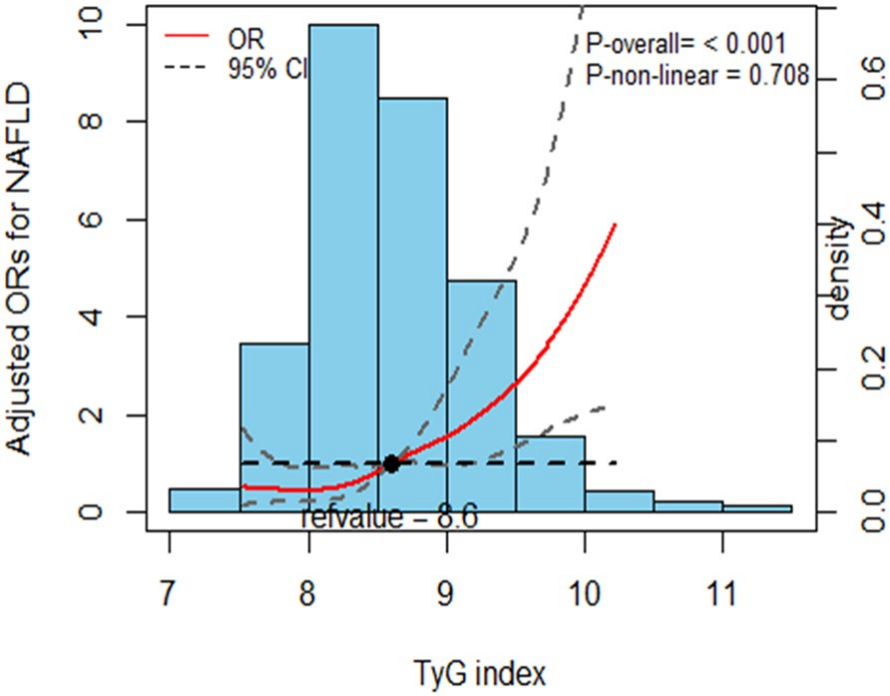
NAFLD prevalence distribution odds ratio and histogram based on TyG index. The red curve with the light black dashed line represents the adjusted odds ratio for the prevalence of NAFLD based on the TyG index, with a 95% CI of 8.6. The cubic spline in the model has 3 knots. Adjustment factors include age, gender, AF type, dyslipidemia, diabetes, BMI, eGFR, AST, ALT, HDL-C, UA, duration of AF, hypertension, and smoking. *NAFLD* non-alcoholic fatty liver disease, *95% CI* 95% confidence interval, *OR* odds ratio, *TyG* triglyceride–glucose, *BMI* body mass index, *eGFR* estimated glomerular filtration rate, *AST* aspartate aminotransferase, *ALT* alanine aminotransferase, *HDL-C* high-density lipoprotein cholesterol, *UA* uric acid

Figure 3 presents the dose–response relationship between the TyG index and NFLD. The result indicated that the OR of the TyG index and NAFLD have a nearly linear relationship (*P* non-linear = 0.71), with the OR of NFLD doubling when the TyG index levels were approximately 8.60.

### Subgroup analysis and sensitivity analysis

**Fig. 4:**
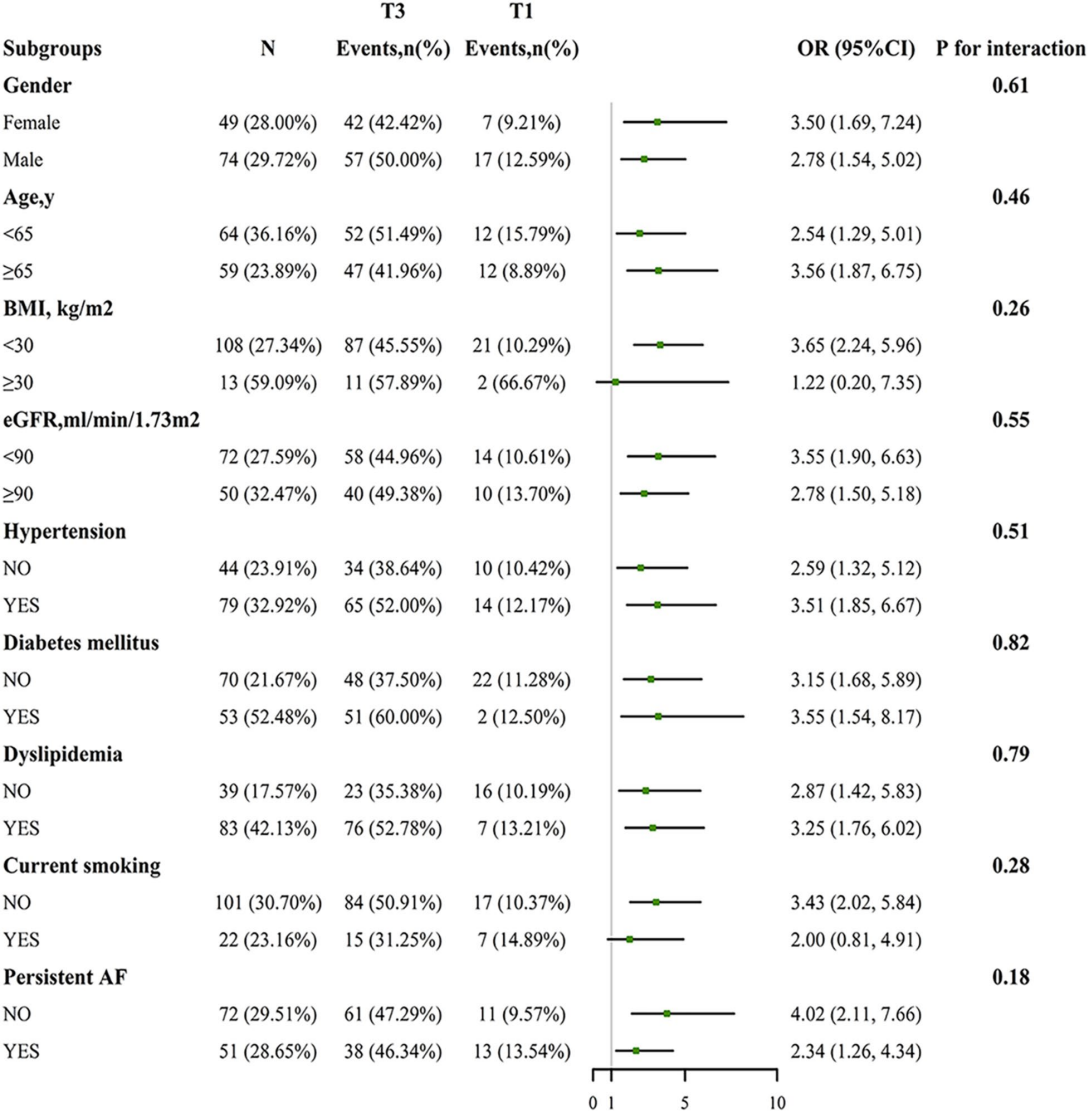
Association between the TyG index (T3 vs. T1) and NAFLD in each subgroups. Adjusted, if not stratified, for age, gender, AF type, dyslipidemia, diabetes, BMI, eGFR, AST, ALT, HDL-C, UA, duration of AF, hypertension, and smoking. *NAFLD* non-alcoholic fatty liver disease, *95% CI* 95% confidence interval, *OR* odds ratio, *TyG* triglyceride–glucose, *BMI* body mass index, *eGFR* estimated glomerular filtration rate, *AST* aspartate aminotransferase, *ALT* alanine aminotransferase, *HDL-C* high-density lipoprotein cholesterol, *UA* uric acid

Subgroup analysis was performed to assess the TyG index associated with NAFLD in predefined subgroups, as shown in Fig. 4. None of the investigated interactions have significance (all *p* interactions > 0.1). Moreover, the sensitivity analysis that included patients with HBV or HCV infection in the overall population to strengthen our results (OR = 2.53; 95%CI 1.54, 4.16) (Additional file 1: Tables S2, S3).

### ORs of the HSI for predicting NAFLD

NAFLD prevalence increased significantly with rising HIS scores (Fig. 2B). The ORs and 95% CIs for tertile 3 of TyG index and HSI were higher than tertile 1 which was 4.97 (95% CI 2.73, 8.40) and 2.52 (95% CI 1.51, 4.21), respectively (Table 3).

**Table 3:** Odds ratios for non-alcoholic fatty liver diseases according to tertiles of the TyG index and HSI

Parameters	Tertile (range)	OR (95% Cl)	*P*
TyG index			
	T1 (≤ 8.29 )	Ref.	1.0
	T2 (8.29–8.82)	2.54 (1.47, 4.39)	< 0.001
	T3 (≥ 8.82)	4.97 (2.73, 8.40)	< 0.001
HSI			
	T1 (≤ 29.26)	Ref.	1.0
	T2 (29.26–33.13)	1.29 (0.77, 2.18)	0.134
	T3 (≥ 33.13)	2.48 (1.44, 4.25)	< 0.001

Adjusted for age, gender, AF type, dyslipidemia, and diabetes

*95% CI* 95% confidence interval, *OR* odds ratio, *TyG* triglyceride–glucose, *HSI* hepatic steatosis index

### Cut-off values and AUC of the TyG index and HSI of predicting NAFLD

**Fig. 5:**
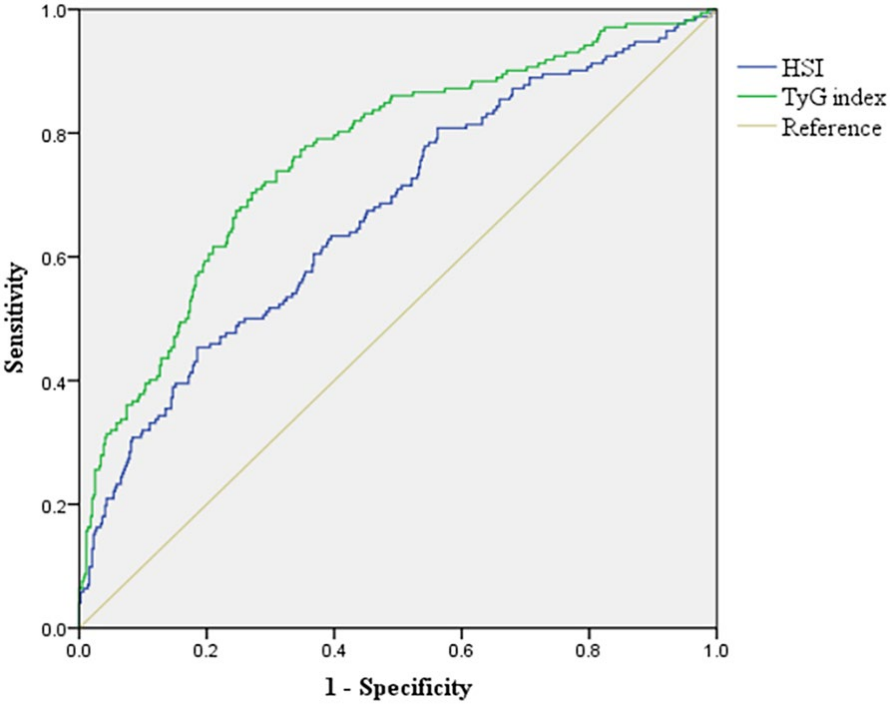
NAFLD Receiver operative characteristic (ROC) curves and corresponding areas under the curve (AUC). *NAFLD* non-alcoholic fatty liver disease, *TyG* triglyceride–glucose, *HSI* hepatic steatosis index

The ROC curves for the TyG index and HSI for predicting NAFLD are presented in Fig. 5. Interestingly, the area under the curve (AUC) of  the TyG index was 0.735 (95% CI 0.690, 0.779), the sensitivity was 0.66, the specificity was 0.72. While the AUC of HSI was 0.677 (95% CI 0.629, 0.725), with a the sensitivity was 0.69, and a the specificity was 0.59 (Table 4). The TyG index and HSI with an  optimal cut-off of 8.6 and 33.9, respectively (Table 4).

**Table 4:** Areas under the ROC curves for each parameter of the TyG index and HSI for predicting NAFLD

Parameters	Cut-off	Sensitivity	Specificity	AUC	95%CI	P
TyG index	8.600	66.010	71.590	0.735	0.690, 0.779	< 0.01
HSI	33.900	69.070	58.720	0.677	0.629, 0.725	< 0.01

*AUC* area under the curve, *TyG* triglyceride–glucose, *HSI* hepatic steatosis index

## Discussion

### Major findings

As we know, there is a NAFLD association with AF. Earlier surveys demonstrated metabolic disorders are a crucial risk factor for NAFLD and AF. However, the TyG index association with the risk of AF patients with NAFLD is unclear. This cross-sectional study demonstrated a significant association between TyG and the risk of NAFLD in patients with AF after adjustment for potential confounders. Stratified analyses illustrated that the results were robust in different population settings. Dose–response analysis indicated that the TyG index value was approximately 8.6, where the NAFLD risk was doubled. Meanwhile, there was a positive association between HSI scores and the risk of NAFLD.

Liver biopsy is the gold standard for the diagnosis of NAFLD, but its disadvantages such as invasiveness, sampling error, and possible complications limit its clinical application. The methods of detecting NAFD include ultrasonography, computed tomography scanning, and magnetic resonance imaging. However, they are time-consuming and expensive [21]. ALT is a common way to detect NAFLD and assess the severity of liver injury, but its capability to identify NAFLD is doubted [22]. A study indicated that a normal blood ALT level was present in 79% of NAFLD patients with a hepatic ultrasound diagnosis [23]. Therefore, the establishment of a more sensitive biomarker to detect NAFLD is necessary.

HSI involves measures including ALT, AST, BMI, gender, and history of diabetes. A study has suggested that HSI is a predictor of NAFLD, with an AUROC 0.812 [24]. Moreover, HSI was associated with a high AUC of 0.929 in Youth [25]. However, in our study, the AUC of HSI to predict NAFLD is 0.677. Compared with previous studies [24, 25], the AUC of HIS in this study is lower, and there are some reasons that may explain this. The enrolled population in the previous study was younger than ours (18.2 vs. 64.8 years old). The major intention of the  present study is to examine the association between the TyG index and the risk of NAFLD in patients with AF. Thus, the individuals with missing glucose and TG information were not enrolled in the final analysis. The formula of the TyG indexis  simpler than that of the HSI is more frequently employed in many studies.

The TyG index originated from fasting plasma glucose and TG, which are key metabolic variables for fatty liver. Meanwhile, the TyG index plays an important role in the development of NAFLD as a surrogate IR marker. Some research indicated that the TyG index is related to metabolic diseases such as diabetes and metabolic syndrome [26, 27]. And the TyG index is a  strongly correlated relationship with the amount of hepatic fat and is a good indicator of hepatic insulin resistance [14]. Recently, the TyG index was applied to identify NAFLD. Rivière, B. et al., found that there was an independent association between the TyG index and NAFLD (OR: 2.0; 95% CI 1.1–3.7) in obese patients [28]. In this study, we found that the TyG index is associated with NAFLD and has a higher AUC of 0.735 to predict NAFLD.

### Comparisons with previous studies

Studies show that there is a remarkable TyG index association with NAFLD among the common population. A cross-sectional study conducted in China, which enrolled 10,761 participants. Subjects in  quartile 4 of the TyG index were more likely to have NAFLD than those in quartile 1, (OR: 6.3; 95% CI 5.3–7.5) after adjustment for age, sex, BMI, SBP, UA, white blood cell count, and ALT quartiles. TyG had an AUC of 0.782 with an optimal cut-off of 8.5. [14]. In a study in which 17,577 subjects were included, Song et al. found that, The OR (95% CI) was 8.656 (7.633-9.817) for NAFLD in quartile 4 of the TyG index compared with participants in quartile 1. TyG had an AUC of 0.773 [15]. A study reported a significant TyG-index association with  NAFLD  among youth. The study had 225 participants aged 10–19 years; subjects in tertile 3 of the TyG index have a higher risk of NAFLD than those in tertile 1  (OR: 8.513; 95% CI 2.424–29.896). TyG had an AUC of 0.761 [25]. Our findings aligned harmoniously with prior investigations, compared to the patients in the lowest tertiles of the TyG index, the OR (95% CI) for NAFLD was 4.15 (2.28, 7.53) in the highest. TyG had an AUC of 0. 735 with an optimal cut-off of 8.6.

### Underlying mechanism

The TyG index is a reliable alternative indicator for IR [29]. The underlying mechanisms of the TyG index relationship with NAFLD could be related to IR. There is a close relationship between IR and NAFLD [12]. First, IR impaired the insulin-sensitive and glucose metabolism of tissue, which caused damage to many organ functions, including the liver and heart [30]. Second, IR has associations with chronic inflammation, which may lead to NAFLD and AF [31, 32]. Last, IR induces oxidative stress to promote stellate cell proliferation and inflammatory liver macrophage activation to cause NAFLD [33].

### Clinical practices

At present, some studies give evidence that a remarkable association between NAFLD and AF [7, 34]. Thus, early  diagnosis of NAFLD may ameliorate the prognosis of AF. This study indicated that the TyG index was positively associated with NAFLD patients with AF, after additional adjustment for age, gender, AF type, dyslipidemia, diabetes, BMI, eGFR, AST, ALT, HDL-C, UA, hypertension, and smoking. In our study, as an IR indicator, the TyG index should be used to highlight the key role of IR in NAFLD in AF patients. Then, the study tried to provide a new method to identification NAFLD in AF patients.

### Limitations

This study has some limitations. First, this was a cross-sectional study and no statements about causality are made. Second, our study had small samples and was single center, which may cause bias. Although we adjusted for confounders in the multivariate analysis, the potential confounders were not completely eliminated. Third, Diagnosis of NAFLD was made by ultrasonography rather than liver biopsy, the gold standard technique for detecting fatty liver. Finally, in this study, we only included AF patients. Therefore, the findings suitable population is limited. Moreover, studies of a large and diverse population should be conducted to further verify. To our knowledge, this is the first study to investigate the association between the TyG index and NAFLD patients with  AF.

## Conclusion

Our findings indicated the TyG index has a significant association with  NAFLD in AF patients. The TyG index may be a good marker for predicting NAFLD in AF patients.

### Supplementary Information

**Additional file 1: Table S1.**. Characteristics by the Tertiles of the triglyceride-glucose index of the study population. **Table S2. **Characteristics of the overall population after including patients with HBV or HCV infection. **Table S3. **Association of the triglycerides-glucose index with risk of nonalcoholic fatty liver diseases after including patients with HBV or HCV infection. **Figure S1. **Prevalence of NAFLD based on the IR. The cut-off for defining IR was set at a TyG index of ≥ 8.76.

## Data Availability

The authors provide without reservation the raw data supporting the conclusions of this paper.

## Abbreviations

TyG: Triglyceride and glucose
NAFLD: Non-alcoholic fatty liver disease
NASH: Non-alcoholic steatohepatitis
IR: Insulin resistance
AF: Atrial fibrillation
ROC: Receiver operating characteristic
ORs: Odds ratios
95% CI: 95% Confidence interval
AUC: Area under the curve
HSI: Hepatic steatosis index
SBP: Systolic blood pressure
DBP: Diastolic blood pressure
BMI: Body mass index
TG: Triglyceride
PTC: Total cholesterol
HDL-C: High-density lipoprotein cholesterol
LDL-C: Low-density lipoprotein cholesterol
HbA1c: Hemoglobin A1c
AST: Aspartate aminotransferase
ALT: Alanine aminotransferase
UA: Uric acid
eGFR: Estimated glomerular filtration rate
SD: Standard deviation
