# Supplementary material for: Association between the Triglyceride–Glucose Index and Non-Alcoholic Fatty Liver Disease in patients with Atrial Fibrillation
Source: Eur J Med Res. 2023 Sep 19;28:355. doi: 10.1186/s40001-023-01188-2 (PMC10507838; doi:10.1186/s40001-023-01188-2)
Supplement: Supplementary file 1 — Additional file 1: Table S1.. Characteristics by the Tertiles of the triglyceride-glucose index of the study population. Table S2. Characteristics of the overall population after including patients with HBV or HCV infection. Table S3. Association of the triglycerides-glucose index with risk of nonalcoholic fatty liver diseases after including patients with HBV or HCV infection. Figure S1. Prevalence of NAFLD based on the IR. The cut-off for defining IR was set at a TyG index of ≥ 8.76. [file 40001_2023_1188_MOESM1_ESM.docx]

**SUPPLEMENTAL MATERIALS**

**Table S1 Characteristics by the Tertiles of the triglyceride-glucose index of the study population.**

**Table S2 Characteristics of the overall population after including patients with HBV or HCV infection.**

**Table S3 Association of the triglycerides-glucose index with risk of nonalcoholic fatty liver diseases after including patients with HBV or HCV infection.**

**Figure S1 Prevalence of NAFLD based on the IR. The cut-off for defining IR was set at a TyG index of ≥ 8.76.**

**Table S1 Characteristics by the Tertiles of the triglyceride-glucose index of the study population.**

| **Characteristics** | **Tertiles of triglyceride-glucose index** | | | **P** |
| --- | --- | --- | --- | --- |
|  | T1 (≤ 8.29)  211 | T2 (8.29-8.82)  210 | T3 (≥ 8.82)  211 |  |
| Age, year | 66.15 (9.26) | 64.50 (9.61) | 63.77 (10.42) | 0.04 |
| Female, n (%) | 76 (36.02) | 96 (45.71) | 97 (45.97) | 0.06 |
| BMI, kg/m2 | 22.96 (2.97) | 24.49 (3.04) | 25.34 (3.63) | < 0.01 |
| Smoke, n (%) | 164 (77.73) | 170 (80.95) | 164 (77.73) | 0.65 |
| Persistent AF, n (%) | 96 (45.50) | 97 (46.19) | 82 (38.86) | 0.25 |
| NAFLD, n (%) | 24 (11.37%) | 53 (25.24%) | 99 (46.92%) | < 0.01 |
| SBP, mmHg | 125 (19) | 126 (19) | 129 (19) | 0.19 |
| DBP, mmHg | 76 (15) | 78 (15) | 76 (14) | 0.28 |
| Duration of AF, months | 30.93 (44.35) | 35.88 (49.66) | 36.13 (51.49) | 0.40 |
| **Laboratory results** |  |  |  |  |
| AST, mmol/L | 23.53 (19.04-28.93) | 23.46 (19.47-28.61) | 23.56 (19.20-28.45) | 0.90 |
| ALT, mmol/L | 18.75 (13.48-27.19) | 19.94 (12.77-27.84) | 21.54 (15.55-30.91) | 0.10 |
| TC, mmol/L | 3.85 (0.92) | 4.21 (1.03) | 4.64 (1.09) | < 0.01 |
| TG, mmol/L | 0.82 (0.69-0.92) | 1.25 (1.11-1.40) | 2.08 (1.67-2.58) | < 0.01 |
| HDL-C, mmol/L | 1.27 (0.35) | 1.16 (0.27) | 1.05 (0.28) | < 0.01 |
| LDL-C, mmol/L | 2.14 (0.70) | 2.51 (0.77) | 2.82 (0.88) | < 0.01 |
| Glucose, mmol/L | 4.67 (4.31-5.12) | 5.12 (4.64-5.62) | 5.91 (5.21-7.21) | < 0.01 |
| HbA1C | 5.70 (5.40-6.00) | 5.70 (5.40-6.00) | 5.90 (5.60-6.50) | < 0.01 |
| eGFR, ml/min/1.73m^2^ | 81.84 (19.38) | 83.07 (21.54) | 84.12 (23.80) | 0.52 |
| UA, mmol/L | 369.26 (102.00) | 374.68 (99.82) | 387.95 (108.88) | 0.17 |
| HSI | 29.80 (4.18) | 31.58 (4.61) | 33.15 (5.24) | < 0.01 |
| TyG index | 7.99 (0.23) | 8.54 (0.15) | 9.30 (0.47) | < 0.01 |
| **Chronic disease, n (%)** |  |  |  |  |
| Hypertension | 115 (54.50%) | 121 (57.62%) | 123 (58.29%) | 0.70 |
| Diabetes | 16 (7.58%) | 32 (15.24%) | 85 (40.28%) | < 0.01 |
| Dyslipidemia | 53 (25.24%) | 71 (34.30%) | 143 (69.08%) | < 0.01 |

The continuous variables are expressed as the mean (SD) for the normally distributed data or the median with an interquartile range for the nonnormally distributed data. The categorical variables are expressed as numbers (percentages). BMI: body mass index; SBP: systolic blood pressure; DBP: diastolic blood pressure; AST: aspartate aminotransferase; ALT: alanine aminotransferase; TC: total cholesterol; TG: triglyceride; HDL-C: High-density lipoprotein cholesterol; LDL-C: Low-density lipoprotein cholesterol; eGFR: Estimated glomerular filtration rate; HbA1C: glycated hemoglobin; UA: uric acid; TyG: triglyceride-glucose; HSI: hepatic steatosis index.

**Table S2 Characteristics of the overall population after including patients with HBV or HCV infection.**

| **Characteristics** | **Total (N = 816)** | **NAFLD (N = 216)** | **Normal (N = 600)** | **P** |
| --- | --- | --- | --- | --- |
| Age, year | 64.26 (10.25) | 62.62 (10.62) | 64.85 (10.05) | < 0.01 |
| Female, n (%) | 374 (45.83) | 80 (37.04) | 294 (39.20) | < 0.01 |
| BMI, kg/m2 | 23.79 (3.19) | 25.07 (3.61) | 23.32 (2.89) | < 0.01 |
| Smoke, n (%) | 156 (19.12) | 38 (17.59) | 118 (19.67) | 0.51 |
| Persistent AF, n (%) | 395 (48.41) | 101 (46.76) | 294 (49.00) | 0.57 |
| SBP, mmHg | 127 (14) | 129 (19) | 127 (19) | 0.18 |
| DBP, mmHg | 76 (14) | 76 (14) | 76 (14) | 0.79 |
| Duration of AF, months | 34.50 (50.74) | 39.40 (55.57) | 32.73 (48.42) | 0.16 |
| **Laboratory results** |  |  |  |  |
| AST, mmol/L | 24.00 (20.00-28.00) | 25.00 (21.00-30.00) | 24.00 (20.00-28.00) | 0.04 |
| ALT, mmol/L | 23.00 (15.75-28.00) | 25.00 (18.00-31.00) | 21.00 (15.00-27.00) | < 0.01 |
| TC, mmol/L | 4.26 (1.08) | 4.61 (1.18) | 4.14 (1.02) | < 0.01 |
| TG, mmol/L | 1.21 (0.90-1.71) | 1.50 (1.09-2.27) | 1.13 (0.83-1.53)) | < 0.01 |
| HDL-C, mmol/L | 1.18 (0.33) | 1.11 (0.30) | 1.21 (0.33) | < 0.01 |
| LDL-C, mmol/L | 2.49 (0.84) | 2.78 (0.92) | 2.38 (0.79) | < 0.01 |
| Glucose, mmol/L | 5.09 (4.56-5.73) | 5.41 (4.80-6.62) | 4.99 (4.51-5.55) | < 0.01 |
| HbA1C | 5.70 (5.40-6.00) | 5.80 (5.50-6.25) | 5.70 (5.30-5.90) | < 0.01 |
| eGFR, ml/min/1.73m^2^ | 84.36 (21.28) | 86.29 (20.82) | 83.67 (21.42) | 0.14 |
| UA, mmol/L | 371.94 (105.43) | 382.24 (104.39) | 368.21 (104.39) | 0.05 |
| HSI | 32.67 (4.87) | 34.66 (5.70) | 31.96 (4.32) | < 0.01 |
| TyG index | 8.57 (0.62) | 8.90 (0.69) | 8.45 (0.55) | < 0.01 |
| **Chronic disease, n (%)** |  |  |  |  |
| Hypertension | 359 (44.00) | 107 (49.54) | 252 (42.00) | 0.56 |
| Diabetes | 149 (18.26) | 66 (30.56) | 83 (13.83) | < 0.01 |
| Dyslipidemia | 298 (36.88) | 116 (53.95) | 182 (30.69) | < 0.01 |
| HBV/HCV | 184 (22.55) | 40 (18.52) | 144 (24.00) | 0.10 |

The continuous variables are expressed as the mean (SD) for the normally distributed data or the median with an interquartile range for the nonnormally distributed data. The categorical variables are expressed as numbers (percentages). BMI: body mass index; SBP: systolic blood pressure; DBP: diastolic blood pressure; AST: aspartate aminotransferase; ALT: alanine aminotransferase; TC: total cholesterol; TG: triglyceride; HDL-C: High-density lipoprotein cholesterol; LDL-C: Low-density lipoprotein cholesterol; eGFR: Estimated glomerular filtration rate; HbA1C: glycated hemoglobin; UA: uric acid; TyG: triglyceride-glucose; HSI: hepatic steatosis index; HBV: hepatitis B virus; HCV: hepatitis C virus.

.

**Table S3 Association of the triglycerides-glucose index with risk of nonalcoholic fatty liver diseases after including patients with HBV or HCV infection.**

| **TyG index** | **Case/N** | **Crude model**  **OR (95%CI)** | **P** | **Model I**  **OR (95%CI)** | **P** | **Model II**  **OR (95%CI)** | **P** |
| --- | --- | --- | --- | --- | --- | --- | --- |
| Per 1 unit increase | 216/816 | 3.44 (2.60,4.56) | < 0.001 | 2.83 (2.01,3.99) | < 0.001 | 2.48 (1.73,3.56) | < 0.001 |
| Tertiles |  |  |  |  |  |  |  |
| T1 (≤ 8.253) | 38/272 | Ref. | 1.0 | Ref. | 1.0 | Ref. | 1.0 |
| T2 (8.253-8.781) | 61/272 | 1.78 (1.14,2.78) | 0.011 | 1.60 (1.01,2.54) | 0.044 | 1.29 (0.79,2.09) | 0.311 |
| T3 (≥ 8.781) | 117/272 | 4.65 (3.06,7.06) | < 0.001 | 3.18 (1.99,5.10) | < 0.001 | 2.53 (1.54,4.16) | < 0.001 |
| P for trend |  | < 0.001 |  | < 0.001 |  | < 0.001 |  |

Note: Crude model was unadjusted for any factors; Model I was adjusted for age, gender, AF type, dyslipidemia, and diabetes. Model II was adjusted for Model I, BMI, eGFR, AST, ALT, HDL-C, UA, duration of AF, hypertension, and smoking.

**
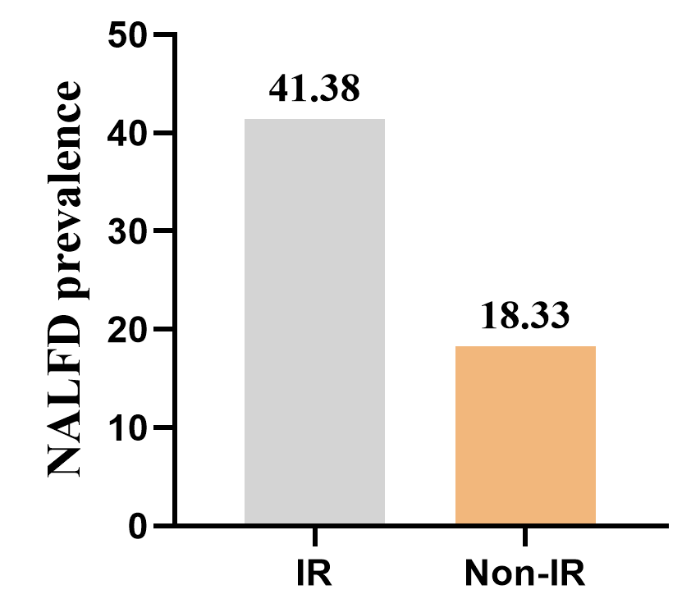
**

**Figure S1** Prevalence of NAFLD based on the IR. The cut-off for defining IR was set at a TyG index of ≥ 8.76.

Abbreviations: NAFLD: nonalcoholic fatty liver disease; IR: insulin resistance; TyG: triglyceride-glucose.
